# Orchidopexy Timing and Follow Up: From Guidelines to Clinical Practice

**DOI:** 10.3390/diagnostics15182318

**Published:** 2025-09-12

**Authors:** Cristina Gavrilovici, Alma-Raluca Laptoiu, Elena Hanganu, Iulia Carmen Ciongradi, Monika Glass, Valentin Munteanu, Anastasia Chirvasa, Ancuta Lupu, Petronela Pirtica, Elena-Lia Spoială, Lucian Boiculese

**Affiliations:** 1 Department of Mother and Child Medicine, Grigore T. Popa University of Medicine and Pharmacy Iasi, 700115 Iasi, Romania; cri.gavrilovici@umfiasi.ro (C.G.); anastasia.chirvasa@gmail.com (A.C.); anca_ign@yahoo.com (A.L.); 2 “Sfânta Maria” Emergency Hospital for Children Iasi, 700309 Iasi, Romania; hanganu.elena1@umfiasi.ro (E.H.); carmen.ciongradi@umfiasi.ro (I.C.C.); valentin.munteanu@umfiasi.ro (V.M.); pp_nela@yahoo.com (P.P.); 3 Department of Biomedical Sciences, Grigore T. Popa University of Medicine and Pharmacy Iasi, 700115 Iasi, Romania; 4 2nd Department of Surgery, Grigore T. Popa University of Medicine and Pharmacy Iasi, 700115 Iasi, Romania; 5 Kannerklinik, Center Hospitalier du Luxembourg, 4 Rue Nicolas Ernest Barblé, 1210 Luxembourg, Luxembourg; glass.monika68@yahoo.com; 6 Biostatistics, Department of Preventive Medicine and Interdisciplinarity, Grigore T. Popa University of Medicine and Pharmacy Iasi, 700115 Iasi, Romania; lboiculese@gmail.com

**Keywords:** undescended testis, orchidopexy, cryptorchidism, risk factors, testicular atrophy

## Abstract

**Background**: Undescended testis (UDT) is the most frequent pediatric anomaly of the male genitals, with a high incidence in premature male neonates. Due to the risk of long-term complications such as infertility, testicular malignancy, and psychological distress, special attention on the accuracy of management is needed. Despite the existence of well-established guidelines recommending early surgical intervention, significant delays in diagnosis, referral, and treatment are still observed in practice. **Objectives**: This study aims to evaluate the clinical management practices of undescended testis at a tertiary pediatric referral center over a ten-year period, with a particular focus on identifying risk factors associated with the development of postoperative testicular atrophy. **Material and Methods**: The following variables were extracted from patient records: the UDT location, age at surgery (we also recorded the mean age per year during the 10 years period), laterality (unilateral or bilateral), associated malformations and comorbidities, family history of UDT in first-degree relatives, type of surgical intervention (open vs. laparoscopic orchidopexy), and imaging diagnosis (ultrasonography, computer tomography). We considered testicular atrophy (TA) as negative outcome after orchidopexy. To identify the variables that independently contribute to the risk of postoperative testicular atrophy, we conducted a multivariate logistic regression analysis. **Results**: A total of 1082 pediatric patients UDT underwent orchidopexy between 2014 and 2023. The median age at surgery was 5.07 years, significantly exceeding current guideline recommendations. TA was observed in 24.8% of cases. Non-palpable testes, higher testicular position (particularly intra-abdominal), associated comorbidities, positive family history, and delayed surgical intervention were identified as independent risk factors for negative outcomes. The multivariate logistic regression model identified the most significant predictors of postoperative testicular atrophy as the presence of comorbidities (associated with more than an eightfold increase in risk), non-palpable testes (3.35 times higher risk compared to palpable ones), a positive family history of undescended testis (approximately 2.7 times higher risk), and older age at surgery, with each additional year of delay increasing the risk by 28.6%. **Conclusions**: Despite the availability of well-established guidelines, significant delays in the diagnosis and treatment of UDT persist in clinical practice. Testicular atrophy remains a relevant postoperative complication, particularly in patients with non-palpable testes, high testicular position, comorbidities, and late surgical intervention.

## 1. Introduction

Undescended testis (UDT) is the most common congenital anomaly of the male genitalia, with a prevalence of approximately 1–4.6% in full-term neonates and up to 45% in premature infants at birth. The prevalence decreases to 1–2% in full-term infants by 6 months of age due to spontaneous testicular descent [1]. Spontaneous descent typically occurs within the first six months of life; beyond this period, spontaneous descent is unlikely, necessitating surgical intervention [2].

Due to the risk of long-term complications such as infertility, testicular malignancy, and psychological distress, timely and accurate management is essential. Current guidelines from the European Association of Urology recommend that orchidopexy be performed between 6 and 12 months of age, and no later than 18 months [3], early intervention being associated with reduced risk of testicular cancer, better fertility outcomes, torsion, trauma, and inguinal hernia and improved testicular growth [4].

There are two main surgical approaches for UDT: traditional open orchidopexy and laparoscopic surgery, both yielding satisfactory outcomes. While the management of UDT has become largely standardized, the treatment of high intra-abdominal testis is controversial and subject to ongoing debate [5]. Complications such as recurrence, scrotal hematoma, wound infection, and vasal injury are relatively rare. However, testicular atrophy (TA) remains one of the most serious adverse outcomes, even when the surgery is performed within the recommended age range [6].

This study aims to evaluate the clinical management practices of undescended testis at a tertiary pediatric referral center over a ten-year period, with a particular focus on identifying clinical and surgical risk factors associated with the development of postoperative testicular atrophy.

## 2. Materials and Methods

We conducted a retrospective study on patients diagnosed with undescended testis (UDT) at the Clinical Emergency Hospital for Children “St. Maria” in Iasi, over a 10-year period (1 January 2014–31 December 2023). Testes were classified as cryptorchid if they were located in a high scrotal, supra-scrotal, or inguinal position, or if they could not be palpated. A retractile testis, however, was regarded as a normal anatomical variation. In this study, testicular atrophy in infants with previously operated UDT was assessed visually and palpably, based on relative size and consistency compared to the contralateral testis. In cases of bilateral UDT, comparison was made to the expected normative features of age-matched testicular size and consistency. Atrophy was defined when the affected testis appeared significantly smaller or softer than the contralateral testis on physical examination or intraoperative inspection, as documented in postoperative follow-up.

The inclusion criteria were pediatric patients diagnosed with undescended testis, who underwent surgery and a postoperative follow-up of minimum 6 months. The exclusion criteria were patients with sexual development disorders, testicular nubbin following neonatal or perinatal testicular torsion, or incomplete follow up/data.

We classified the UDT according to the 2024 European Association of Urology into: palpable testes (typically located within the inguinal canal) and non-palpable (intra-abdominal or ectopic (suprapubic, femoral, suprascrotal, or perineal positions).

The following variables were extracted from patient records: the UDT location, age at surgery (we also recorded the mean age per year during the 10 years period), laterality (unilateral or bilateral), associated malformations and comorbidities, family history of UDT in first-degree relatives, type of surgical intervention (open vs. laparoscopic orchidopexy), and imaging diagnosis (ultrasonography, computer tomography). We considered testicular atrophy as negative outcome after orchidopexy. Testicular atrophy was assessed by visual inspection and palpation, comparing the affected testis with the contralateral normally descended testis when available. This method, while subjective, reflects standard clinical practice in our setting where routine ultrasound measurement of testicular volume is not performed. We acknowledge that ultrasonography would have provided more objective measurements; however, this was not feasible in our cohort.

Comorbidities were defined as associated medical conditions present at the time of diagnosis or surgery. These included neurological disorders (e.g., cerebral palsy, developmental delays), genitourinary malformations (such as hypospadias or ectopic kidneys), chromosomal or genetic syndromes (e.g., Down syndrome), severe prematurity with neonatal complications, and other chronic illnesses (e.g., congenital heart defects or rare metabolic diseases).

All data were analyzed using SPSS software version 17.0. Statistical methods included: univariate analysis to evaluate the potential risk factors for TA development: prematurity, comorbidities, family history, age at surgery, testicular location, laterality, associated hernia, and type of surgery. To identify the variables that independently contribute to the risk of postoperative testicular atrophy, we conducted a multivariate logistic regression analysis. This statistical method allowed us to control for potential confounding factors and to isolate the individual effect of each variable—such as testicular location, age at surgery, presence of comorbidities, and family history—on the likelihood of an unfavorable outcome.

The study protocol was reviewed and approved by the Ethical Committees of “Grigore T. Popa” University of Medicine and Pharmacy, Iasi, Romania (No.313/29.05.2023) and of “St Maria” Clinical Emergency Hospital for Children, Iasi (No. 34711/26.11/2021).

## 3. Results

A total of 1082 patients with UDT were included in the study. Sample characteristics regarding laterality, classification, testicular location, comorbidities, and family history are detailed in Table 1.

We performed a multivariate logistic regression with five clinical variables: testicular classification (palpable vs. non-palpable), age at surgery, presence of an associated inguinal hernia, positive family history of UDT and the presence of associated malformations or comorbidities (Table 2).

The median age at diagnosis was 5.07 years (range: 0.5–17 years), the same like the overall median age at the time of surgery. The median age at surgery was slightly lower for patients with bilateral UDT (4.58 years) than for those with unilateral cases (4.67 years).

**Figure 1:**
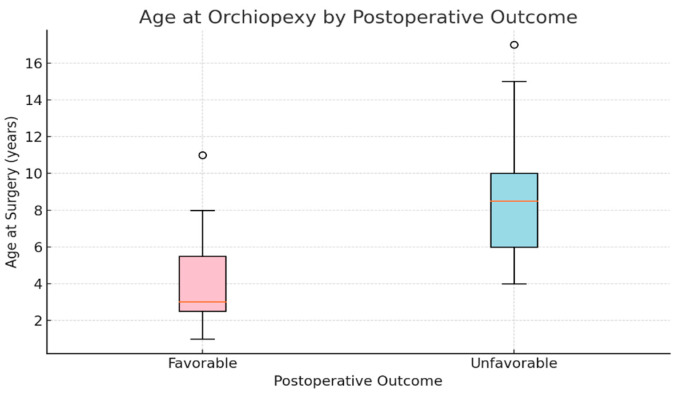
Age at Orchidopexy According to Postoperative Outcome: Favorable vs. Unfavorable Evolution.

**Figure 2:**
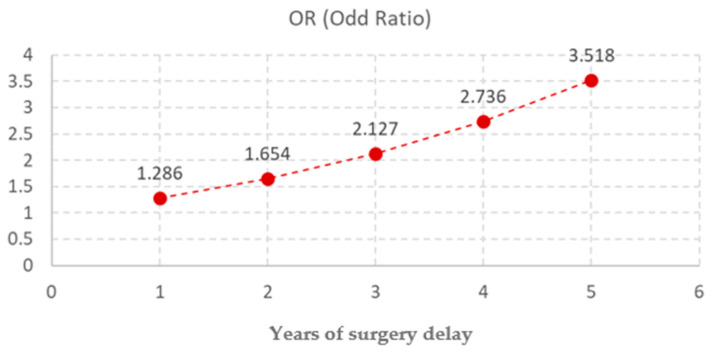
Odds Ratios of Independent Risk Factor for Postoperative Testicular Atrophy Following Orchidopexy.

Age at surgery was a strong and statistically significant predictor (*p* < 0.001) for the negative outcome. The median age for the favorable outcome was 3 years old while the median age for the unfavorable was 8 years old (Figure 1). Each additional year of age increased the odds of a negative outcome by approximately 29% (OR = 1.286, 95% CI: 1.234–1.340), supporting the importance of early intervention (Figure 2)

**Figure 3:**
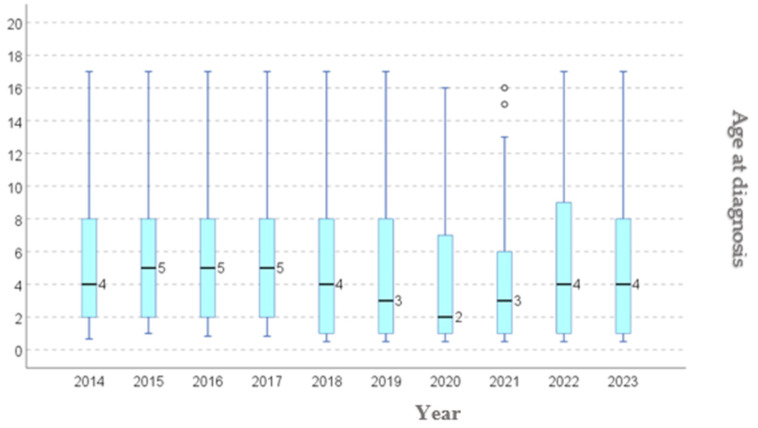
Age at diagnostic: distribution per year.

We conducted a year-by-year analysis of the age at diagnosis among the patients included in this study, to assess the age trend, and whether if this develops in alignment with the international guidelines. From 2019, an initial declining trend in the median age was observed, from 5.39 (in 2014) years to 4.03 years old (in 2021) (Table 3, Figure 3), followed by an increase in age up to 5.05 in 2023.

Most cases involved unilateral UDT (78.8%), while bilateral involvement was observed in 21.2% of patients. However, the difference was not statistically significant. The data indicate that the postoperative outcomes are similar regardless of laterality, with positive results occurring in 75.5% of bilateral cases and 75.1% of unilateral cases. These findings suggest that laterality does not significantly influence postoperative prognosis in patients undergoing orchidopexy (Table 4).

Palpable testes were more frequently diagnosed, comprising 92.2% of the cases, whereas non-palpable testes represented only 7.8%. The odds ratio (OR) of 0.298 (palpable versus non-palpable UDT) suggests that palpable testes are associated with a lower risk of negative outcomes. 1/0.298 = 3.35: patients with non-palpable UDT have a 3.35-fold higher risk of experiencing a negative outcome, such as TA, compared to those with palpable testes. These results highlight that a high testicular location, particularly intra-abdominal testes, is an independent and significant risk factor for testicular atrophy following orchidopexy (Table 2).

Comorbidities were present in 11.7% of the children, while 88.3% had no associated conditions. Additionally, a positive family history of UDT was reported in 11.5% of patients. Patients presenting with comorbid conditions have a significantly increased risk of unfavorable postoperative outcomes following orchidopexy. Specifically, the odds of experiencing an adverse evolution are 2.72 times higher in patients with comorbidities compared to those without, with an odds ratio (OR) of 8.26. The range of comorbidities considered in this analysis included a variety of congenital and systemic conditions such as abdominal wall defects, cardiac anomalies, cleft lip and palate, renal anomalies (including renal agenesis), as well as genetic syndromes like Down syndrome and Noonan syndrome. These comorbidities likely contribute to increased surgical complexity and may affect both the vascular supply and healing capacity of the testicular tissue, thereby influencing postoperative outcomes.

The positive family history was significantly associated with an increased risk of postoperative complications (OR = 2.720, 95% CI: 1.707–4.334, *p* < 0.001). This implies that children with a familial background of UDT are over 2.7 times more likely to experience a negative outcome, emphasizing a potential genetic or biological vulnerability. In our study, 3.7% of patients were associated with prematurity. However, there is no significant impact on testicular atrophy (Table 2).

The presence of an associated inguinal hernia had a modest but statistically significant positive effect (OR = 0.640, 95% CI: 0.414–0.991, *p* = 0.045), suggesting that children with hernias might benefit from earlier or more prompt diagnosis and surgery, possibly explaining the better outcomes. In our sample, children without hernia were therefore 1.56 times more likely to have poor outcomes (Table 2).

## 4. Discussion

Testicular descent usually occurs within the first three months of life. A total of 82% of spontaneous descent happened before 2 months of age, although re-ascent may occur in some cases later. Spontaneous descent beyond 6 months is rare. According to Wenzler et al. [2], only 6.9% of UDT cases achieve descent between 6 and 12 months, and fairly no spontaneous descent occurs in those presenting after 6 months. According to European Association of Urology Guidelines on Paediatric Urology [7] if the testis remains undescended by 6 months corrected age, spontaneous descent is highly unlikely and surgical referral is advised. Cryptorchidism is associated with disrupted hormonal signaling and impaired germ cell development, which underlies the increased risk of TA and infertility [8].

We assessed a cohort of 1082 boys with UDT with a minimum follow up of 6 months with the main purpose of underlying the characteristics and associated risk factors for a negative outcome. Given that surgical correction is typically undertaken after the age of 6 month, it is essential to evaluate associated postoperative outcomes—most notably TA, which remains a major clinical concern.

Consensus guidelines—including those from the European Association of Urology and American Urological Association—recommend performing orchidopexy between 6–18 months of age, ideally before one year, to optimize testicular development and reduce risk of long-term sequelae [9]. In our year-by-year analysis, the median age at diagnosis decreased from 5.39 years in 2014 to 4.03 years in 2021, followed by a rebound to 5.25 years in 2023 (Table 2, Figure 1). We attribute this initial decline to disruptions in standard healthcare pathways during the COVID 19 pandemic, which likely altered referral timing and elective surgery scheduling. Consequently, our cohort exhibits a relatively advanced age at surgical intervention, exceeding recommended timeframes. This pattern echoes global trends: recent data indicate that during the pandemic, elective surgical procedures experienced significant delays, including orchidopexy. For instance, in the US, a reduced proportion of children received timely orchidopexy in the COVID era, when much of the focus has been on pediatric emergencies and delays in UDT referrals and surgery occurred, due to lockdowns and resource reallocations [4].

However, even in the pre pandemic period, many children in Western countries still undergo surgery at median ages significantly older than one year—often between 4 and 5 years of age—due to delayed referral, variable guideline adoption, or secondary descent [4]. Thus, this trend is not unique to our cohort. Several studies from both high- and middle-income countries have reported a higher-than-recommended age at orchidopexy, even in the absence of pandemic-related disruptions [6]. Hrivatakis et al. [6] describe in a retrospective multicenter analysis the mean age at surgery ranging from 42 to 67 months—well beyond the preferred window of ≤18 months. Furthermore, data from ORCHESTRA study [10] indicate that only 15% of hospital-treated and 5% of outpatient-treated boys received surgery before their first birthday, despite the National German guideline recommending orchidopexy by 12 months of age [11]. In the United States, the State Ambulatory Surgery Database (2012) and Paediatric Health Information System (2015) indicated a median age at repair of 4 years with 64% of boys having surgery after age 2, and a median age rising to 5 years in the PHIS cohort—with nearly 70% of cases occurring beyond the guideline-recommended period [12]. Similarly, Chinese cohort data and broader analyses have shown a gradual reduction in median orchidopexy age from 3 to 2 years between 2010 and 2015, yet still well above guideline targets of under 18 months [13]. In none of those years did the median age meet the recommended 6–9-month window. These findings underscore a persistent delay in the timing of orchidopexy and reflect systemic issues in referral practices, guideline adoption, and awareness among primary healthcare providers.

In our cohort, most cases involved unilateral UDT (78.8%), while bilateral involvement was observed in 21.2% of patients. This distribution aligns with current epidemiological data: unilateral UDT is observed in approximately two-thirds of cases, with bilateral involvement accounting for about 10–30% of presentations, emphasizing that unilateral presentations are more common. However, we did not find a significant impact of uni/bilaterality on the post-surgery outcome. This finding is consistent with existing evidence: in a large cohort study evaluating over 1600 children, laterality was not an independent predictor of testicular atrophy (*p* ≈ 0.49, OR 0.93, 95% CI 0.75–1.15) [14]. Their model demonstrated that age at operation, prematurity, and laterality did not significantly alter the incidence of TA, underscoring that anatomical and procedural variables carry more prognostic weight. These findings reinforce that, bilateral involvement by itself does not portend worse outcomes after orchidopexy—and supports the validity of our own observation [15]. Collectively, these results suggest that the number of affected testes does not substantially modify the postoperative prognosis and should not alter surgical planning or timing recommendations.

The rates of unfavorable outcomes are likewise comparable (24.5% for bilateral vs. 24.9% for unilateral). These findings suggest that laterality does not significantly influence postoperative prognosis in patients undergoing orchidopexy. A 2019 retrospective cohort study (230 UDTs) specifically analyzed predictors for testicular atrophy [15]. Multivariate analysis found that factors such as primary testicular position (e.g., above inguinal vs. canalicular) and congenital/endocrine disorders—but not laterality, age, or prematurity—were associated with post operative atrophy. Laterality’s stronger association is with fertility and endocrine function—not atrophy. However, it is beyond the purpose of our research to study the fertility rates in our sample.

In our cohort, palpable testes were more frequently diagnosed and were associated with a significantly lower risk of negative postoperative outcomes, whereas non-palpable undescended testes had a 3.35-fold higher risk of complications such as testicular atrophy. This observation aligns with existing literature indicating that higher testicular location, especially intra-abdominal testes, is an independent and substantial risk factor for post orchidopexy TA [16]. Yang and colleagues developed a prediction model based on over 1600 cases of pediatric orchidopexy, identifying that higher testicular location was significantly associated with increased risk of TA (OR ≈ 1.90; *p* = 0.001), whereas laterality and age at surgery were not independent predictors [16]. Moreover, a separate multivariate analysis found that inguinal and above-inguinal testicular locations were associated with TA (hazard ratio 11.76; 95% CI: 1.55–89.33; *p* = 0.017), whereas variables such as age at operation, prematurity were not significant contributors [17]. Non-palpable testes are more likely to be located intra-abdominal or high inguinal, with higher risk of vascular compromise, longer spermatic cords, more hypoplastic or dysgenetic [18].

A positive family history of UDT was identified as an independent predictor of unfavorable postoperative outcomes, with an odds ratio (OR) of 2.72. This finding suggests a nearly threefold increased risk of developing testicular atrophy in patients with affected first-degree relatives compared to those without familial history. Earlier studies, such as those by Schnack et al. [19] and Sijstermans et al. [20], described familial clustering of UDT primarily as a risk factor for the occurrence of the condition, without focusing on postoperative prognosis. These studies, based on smaller cohorts, highlighted that up to 10–15% of UDT cases reported familial aggregation, particularly involving fathers and brothers [19,20]. However, they did not specifically analyze whether familial predisposition influenced surgical outcomes like TA. More recent large-scale analyses have addressed this gap. Yang et al. [15], analyzing over 1600 cases, included family history in their multivariate prediction model for testicular atrophy and confirmed its independent predictive value. Specifically, they reported that children with a positive familial background had a significantly higher risk of postoperative testicular atrophy, with an odds ratio of 2.53 (95% CI: 1.62–3.94, *p* < 0.001), even after adjusting for factors such as testicular location and comorbidities. This reinforces the notion that hereditary predisposition not only increases the likelihood of UDT occurrence but also plays a direct role in determining surgical outcomes, supporting the relevance of incorporating family history into clinical risk assessment protocols [21]. Our results align with this updated perspective, reinforcing that detailed family history should be systematically collected during patient evaluation. In clinical practice, identifying familial risk allows for tailored management, including prioritizing earlier surgery and closer postoperative monitoring in high-risk patients.

In our study, prematurity was not identified as an independent predictor of unfavorable postoperative outcomes following orchidopexy. Several studies, including the one by Aljadani et al., have indicated that prematurity and low birth weight are linked to a higher incidence of undescended testis and may predispose patients to postoperative testicular atrophy, likely due to impaired germ cell development in the preterm testis [22]. Although gestational age at birth was recorded for all patients, its association with testicular atrophy did not reach statistical significance in either univariate or multivariate analyses. This finding is in line with results reported by Tseng et al. [15] who found no significant impact of prematurity on postoperative testicular atrophy in their cohort of 182 boys with congenital undescended testes. Similarly, Sijstermans et al. [20] emphasized that while prematurity is a known risk factor for the occurrence of UDT—due to incomplete descent at birth—it does not appear to influence surgical prognosis once the testis has been surgically corrected. These observations suggest that although preterm birth contributes to the pathogenesis of UDT, it does not represent a determinant of long-term testicular viability post-orchidopexy. This distinction is clinically relevant, as it implies that surgical planning and risk assessment should focus primarily on anatomical and familial factors rather than gestational age history [23].

In our study, timing of orchidopexy emerged as a key independent predictor of postoperative outcome, with each additional year of delay associated with a 28.6% increase in the risk of unfavorable evolution (OR = 1.286; *p* < 0.001). Despite broad international consensus recommending surgery between 6 and 18 months of age, the median age at surgery in our cohort was 5.07 years, reflecting a persistent delay in referral and intervention. These results are consistent with several large cohort studies. Braga et al. [24], in a prospective analysis of 482 boys, reported significantly higher rates of testicular atrophy and reduced testicular growth in children operated after 18 months compared to those treated earlier. While earlier studies, such as those by Sijstermans et al. [20], focused on the epidemiology of delayed surgery, more recent evidence emphasizes its direct prognostic impact. The biological rationale is also well-established: prolonged exposure of the testis to higher intra-abdominal or inguinal temperatures disrupts germ cell development and vascular integrity, increasing vulnerability to atrophy [25]. Non-palpable cryptorchidism still poses challenges and protecting blood flow is essential, given its role in preventing testicular atrophy [26]. Our findings, in concordance with these reports, reinforce that delayed surgery is not just a matter of policy compliance, but a modifiable risk factor with tangible consequences for surgical success and long-term testicular health. Addressing delays through improved awareness among general practitioners and caregivers should therefore be a priority in optimizing the management of cryptorchidism. As we previously reported, achieving a correct and comprehensive diagnosis of cryptorchidism requires the medical team to decide on the appropriate imaging studies, reinforcing the importance of preoperative ultrasound in risk stratification and surgical planning, especially in cases of intraabdominal testes [27].

In our cohort, patients with one or more comorbidities had an eightfold increase in risk of developing testicular atrophy. These findings are in strong agreement with those of Hosseinpour et al., who reported a 4- to 9-fold higher risk of testicular atrophy associated with diverse comorbidities—such as chromosomal syndromes, neurological disorders, and genitourinary malformations—in a cohort of 70 children [28]. Similarly, Yang et al. [16], analyzing over 1600 pediatric orchidopexy cases, identified comorbid conditions as independent predictors of testicular atrophy (OR ≈ 3.7), after adjusting for age, location, and family history. Additionally, a nationwide registry-based study by Braga et al. [24] involving more than 800 cases found that patients with comorbidities experienced a twofold higher rate of surgical failure, defined by compromised testicular growth or reintervention necessity. These converging data highlight that comorbidities consistently emerge as one of the most potent predictors of adverse postoperative outcomes across diverse populations and settings. The underlying mechanisms may include impaired vascularization, endocrine dysregulation, or surgical delays caused by preexisting health conditions. Meanwhile there are also studies, such as the one conducted by the Paediatric Surgical Trainees Research Network, reporting that the timing of orchidopexy does not significantly influence the rate of TA [10]. A randomized clinical trial assessing testicular growth after orchidopexy showed that boys who underwent surgery at 9 months experienced significantly greater testicular development by age 4 compared to those operated at 3 years 24 [22]. Given the indirect relationship between testicular volume and spermatogenic function, these findings suggest that earlier intervention may improve future fertility potential.

Our findings support the classification of patients with comorbidities as a high-risk group, requiring early referral, individualized surgical planning, and close postoperative monitoring to reduce the elevated risk of testicular atrophy. This recommendation is consistent with recent evidence showing that comorbidities independently contribute to delays in orchiopexy, which are in turn linked to less favorable surgical outcomes and an increased risk of complications [29].

In our study, the presence of an inguinal hernia was associated with better outcomes (modest but statistically significant). This intriguing result may be explained by the fact that hernias are visible and symptomatic—they may prompt quicker medical attention, and this might lead to faster diagnosis of the primary condition, reducing delays that are often a major contributor to poor outcomes. In fact, as Elseth et al. emphasized, more than 90% of cases with undescended testis present a patent processus vaginalis or associated inguinal hernia, highlighting how early clinical symptoms can improve referral timing and influence prognosis [18]. The American Urological Association recommends that surgical correction of cryptorchidism should occur between 6 and 18 months of age to optimize fertility potential and reduce the risk of malignancy [9].

Future studies could benefit from examining additional potential risk factors for testicular re-ascent, such as the surgeon’s level of experience, the child’s body mass index, a more precise anatomical classification of the undescended testis, and an objective assessment of the tension within the spermatic cord structures [30]. Given that orchidopexy is among the most frequently performed pediatric surgical procedures, even modest enhancements in surgical technique could lead to meaningful improvements for patients and families, as well as contribute to cost reduction [31].

## 5. Conclusions

This study offers important insights into the management and outcomes of UDT, with a particular focus on the following risk factors for an unfavorable outcome, specifically postoperative TA: familial predisposition, timing of intervention, testicular laterality, associated comorbidities, testicular classification (palpable versus non-palpable), anatomical location of the testis (inguinal, ectopic, or intra-abdominal), and prematurity. By identifying these risk factors, this study contributes to improving patient stratification and tailoring surgical strategies in UDT management, offering valuable guidance for both clinical practice and future research directions.

According to the present data, age at surgery, testicular classification, anatomical location, presence of comorbidities, and family history represent independent predictors of unfavorable postoperative outcomes following orchidopexy for undescended testis. The risk of testicular atrophy increases progressively with surgical delay, reinforcing the need for early intervention. Non-palpable and intra-abdominal testes are significantly more prone to atrophy, while comorbidities remain the strongest negative prognostic factor identified. Familial predisposition also contributes to postoperative risk and should be taken into account during patient assessment. In contrast, testicular laterality and prematurity did not influence postoperative evolution, while the presence of an associated inguinal hernia was correlated with improved outcomes, possibly due to earlier referral. These findings support a comprehensive, risk-adapted approach in the management of cryptorchidism, with emphasis on early diagnosis and individualized follow-up strategies.

Our findings emphasize a persistent delay in the timing of orchidopexy. Therefore, changes in the referral practices, guideline adoption, and increased awareness among primary healthcare providers and pediatricians are paramount. Given that a family history of cryptorchidism may be associated with less favorable outcomes, it is important that primary care providers actively inquire about this during routine wellness checks. Early referral of children with a positive family history should be encouraged to optimize management and prognosis.

## Figures and Tables

**Table 1:** Main characteristics of the sample.

Variable	No. (%)	Median Age (Years)	Negative Outcome, No (%)
**Laterality**			
Unilateral	853 (78.8%)	4.67	209 (24.5%)
Bilateral	229 (21.2%)	4.58	55 (24.9%)
**Classification**			
Palpable	998 (92.2%)	4.75	238 (23.8%)
Non-palpable	84 (7.8%)	5.35	30 (35.7%)
**Testicular location**			
Inguinal canal	943 (87.2%)	5.07	217 (23.0%)
Ectopic	56 (5.2%)	6.73	14 (25.0%)
Intra-abdominal	83 (7.7%)	5.25	32 (38.6%)
**Comorbidities**			
Yes	127 (11.7%)	5.17	83 (65.4%)
No	955 (88.3%)	5.38	180 (18.8%)
**Family History**			
Positive	124 (11.5%)	6.00	58 (46.8%)
Negative	958 (88.5%)	4.85	210 (21.9%)
**Total**	1082 (100%)	5.07	268 (24.8%)

**Table 2:** Predictors of Negative Outcome after Orchidopexy.

Variable	*p* Value	Exp(B) (OR)	95% CI Lower	95% CI Upper
**Palpable vs. non-palpable UDT**	<0.001	0.298	0.171	0.520
**Laterality**	0.611	1.116	0.730	1.707
**Age at Surgery**	<0.001	1.286	1.234	1.340
**Associated Hernia**	0.045	0.640	0.414	0.991
**Family History**	<0.001	2.720	1.707	4.334
**Prematurity**	0.824	0.897	0.343	2.346
**Associated Malformations**	<0.001	8.268	5.246	13.031

**Table 3:** Median age at surgery during the research period 2014–2023.

		Year									
		2014	2015	2016	2017	2018	2019	2020	2021	2022	2023
Mean		5.3909	5.3231	5.4772	5.3889	5.1660	4.8138	4.1373	4.0316	5.3052	5.0586
95% Confidence Interval for Mean	Lower Bound	4.6309	4.6438	4.7309	4.6810	4.4120	3.9613	2.8823	3.1626	4.2972	4.3103
	Upper Bound	6.1510	6.0023	6.2235	6.0968	5.9201	5.6663	5.3923	4.9007	6.3132	5.8068
5% Trimmed Mean		5.0315	5.0214	5.1415	5.0772	4.8013	4.4475	3.7042	3.6593	4.9737	4.7717
**Median**		**4.0000**	**5.0000**	**5.0000**	**5.0000**	**4.0000**	**3.0000**	**2.0000**	**3.0000**	**4.0000**	**4.0000**
Variance		19.787	15.321	16.610	15.728	19.178	20.539	19.911	15.053	19.725	18.302
Std. Deviation		4.44821	3.91423	4.07549	3.96580	4.37931	4.53197	4.46215	3.87988	4.44125	4.27804
Minimum		0.67	1.00	0.83	0.83	0.50	0.50	0.50	0.50	0.50	0.50
Maximum		17.00	17.00	17.00	17.00	17.00	17.00	16.00	16.00	17.00	17.00
Range		16.33	16.00	16.17	16.17	16.50	16.50	15.50	15.50	16.50	16.50
Interquartile Range		6.50	6.00	6.00	6.00	7.00	7.00	6.00	5.00	8.00	7.00
Skewness		0.900	0.806	0.878	0.819	0.955	1.013	1.275	1.351	0.800	0.862
Kurtosis		−0.090	0.179	0.351	0.170	−0.008	−0.057	0.554	1.080	−0.188	−0.247

**Table 4:** Testes Laterality in relation to post-surgery outcome.

						Post Surgery Outcome			Total
						Favorable		Unfavorable	
Laterality	Bilateral		Count			173		56	229
			%			**75.5%**		**24.5%**	100.0%
	Unilateral		Count			641		212	853
			%			**75.1%**		**24.9%**	100.0%
Total			Count			814		268	1082
			%			75.2%		24.8%	100.0%
**Chi-Square Tests**									
		Value		Df	**Asymptotic Significance (2-sided)**		Exact Sig. (2-sided)		Exact Sig. (1-sided)
Pearson Chi-Square		0.015		1	**0.901**				
Continuity Correction		0.001		1	0.970				
Likelihood Ratio		0.015		1	0.901				
Fisher’s Exact Test							0.931		0.488
No of Valid Cases		1082							

## Data Availability

The data presented in this study are available on request from the corresponding author due to privacy.
